# The Epigenetic Landscape of Promoter Genome-wide Analysis in Breast Cancer

**DOI:** 10.1038/s41598-017-06790-z

**Published:** 2017-07-26

**Authors:** Seher Karsli-Ceppioglu, Aslihan Dagdemir, Gaëlle Judes, André Lebert, Frédérique Penault-Llorca, Yves-Jean Bignon, Dominique Bernard-Gallon

**Affiliations:** 1 Department of Oncogenetics, Centre Jean Perrin, CBRV, 28 place Henri-Dunant, 63001 Clermont-Ferrand, France; 2 INSERM U 1240, IMOST, 58 rue Montalembert-BP184, 63005 Clermont-Ferrand, France; 3 Department of Toxicology, Faculty of Pharmacy, Marmara University, Istanbul, Turkey; 4 University Blaise Pascal, Institute Pascal UMR 6602 CNRS/UBP, 63178 Aubiere, France; 5 Department of Biopathology, Centre Jean Perrin, 58 rue Montalembert, 63011 Clermont-Ferrand, France

## Abstract

Breast cancer is a heterogeneous disease due to its clinico-pathological features and response to therapy. The classification of breast tumors based on their hormone receptor status and pathologic features. Post-translational histone modifications come into prominence for regulation of gene expression in cancer pathogenesis. Here, we analyzed dysregulation of H3K9ac and H3K27me3-enriched subtype-specific genes using ChIP-on-chip assay in breast cancer tumors and matched normal tissue samples. Breast cancer tumors were classified according to St Gallen Consensus 2013. Our results indicated that the promoter regions of genes modified by H3K9ac epi-mark are commonly associated with tumors with HER2-positive and TNBC subtype. H3K27me3-enriched genes were comprised of Luminal A and B1 subtypes. We constructed a network structure to elicit epigenetically regulated genes related with breast cancer progression. The central genes of the network (*RUNX1*, *PAX3*, *GATA4* and *DLX5)* were subjected for epigenetically dysregulation in association with different breast cancer subtypes. Our study submits epigenetic mechanisms are crucial to elicit subtype-specific regulation in breast cancer and ChIP-on-chip assay provides a better understanding for breast tumorigenesis and new approaches for prevention and treatment.

## Introduction

Breast cancer is a highly heterogeneous disease due to its clinico-pathological features and response to therapy. Breast tumors are mainly classified into ER-positive, HER2-positive and triple-negative breast cancer (TNBC) based on hormone receptor status^1^. ER and HER2-positive tumors were found to have better five-year relative survival, good prognosis and responsive to hormone therapy^2^. HER2-amplified tumors (overexpress ERBB2)^3^ also show good clinical outcome through therapeutic targeting of HER2^4^. TNBC tumors are commonly basal-like originated from breast epithelial stem cells. Five-year relative survival of TNBC was found lower than women with other breast cancer subtypes. These tumors have poor prognosis and are prone to metastasis^2^.

St Gallen International Expert Consensus focuses on developments in early breast cancer therapy. Since 1978, it has been held routinely and the agreed recommendations are being publishing every two years. A new system for breast cancer classification was propounded in 2011 Conference^5^ and the criteria to identify subtypes were updated in 2013^6^. According to St Gallen Conference 2013, breast cancer subtypes are classified as; luminal A (ER and PGR-positive, low rate of Ki-67 and HER2-negative), luminal B1, HER2-negative (ER-positive, PGR <10% or negative, high rate of Ki-67), luminal B2 HER2-positive (ER-positive and PGR-negative), HER2-positive non-luminal (ER and PGR-negative) and basal-like (ER, PGR and HER2-negative). Since immunohistopathological features vary in luminal disease, luminal subtype is subdivided into Luminal A, B1 and B2^6^. Although Luminal A tumors have favorable prognosis, tumors with Luminal B subtype are more aggressive and the percentage of lymph node involvement was observed higher than women with Luminal A tumors. Luminal B1 subtype differs from Luminal A due to higher levels of Ki67, a nuclear marker of cell proliferation^7^. Lymph node involvement was found to be higher due to increased expression level of Ki67 antigen^8^. Ki67 index is prominent marker to indicate tumor aggressiveness, hence cell proliferation activity increases in Luminal B, HER2-positive and TNBC tumors^7, 9^. Different multi-gene assays for analysis gene expression patterns in breast cancer provide prognostic information, develop prediction models and increase the accuracy of breast cancer subtype classification^4, 10^.

A large number of studies have focused on genetic basis of breast cancer, whereas recently knowledge about the impact of epigenetic mechanisms on breast cancer development and prognosis has been increasing^11^. Post-translational histone modifications are involved in regulation of gene expression in cancer pathogenesis. Furthermore, diversity of breast cancer subtypes are linked to dysregulation of gene expression associated with histone modifications^12^. To our best knowledge, investigations on modifications in breast cancer tumors are very limited.

Nowadays, chromatin immunoprecipitation (ChIP) is coupled with promoter DNA microarrays to evaluate the mechanisms of human gene regulation on a genome-wide scale. ChIP-on-chip technology could be used to investigate the alterations of global gene expression in tumorigenesis. In this study, we aimed to elicit differently regulated genes, associated with modified histone 3 lysine 27 trimethylation (H3K27me3) and histone 3 Lysine 9 acetylation (H3K9ac) in breast cancer tumors by ChIP-on-chip method. For this purpose, Agilent SurePrint G3 400k Human Promoter Microarrays were used to scan gene promoters in 15 breast tumors with their matched normal tissue samples. Breast tumor samples were classified according to St Gallen Consensus 2013 to identify the impact of epigenetic patterns on the diversity of breast cancer subtypes.

## Results

### Classification of Breast Cancer Subtypes

Breast cancer tumor samples have been subdivided into five molecular subtypes according to St Gallen International Expert Consensus 2013: luminal A (ER and PGR-positive, human Ki-67 protein <14%, histological grade 1 or 2 and HER2-negative), luminal B1, HER2-negative (ER-positive, PGR <10% or negative, Ki-67 > 14% and high grade), luminal B2 HER2-positive (ER-positive and PGR-negative), HER2-positive non-luminal (ER and PGR-negative) and TNBC (ER, PGR and HER2-negative). St Gallen classification is based on clinic-pathological factors of early invasive breast cancer. Luminal disease is responsive to endocrine therapy and usually has a more favorable prognosis. TNBC and HER2-positive diseases have poor clinical outcome. On the other hand, Luminal A subtype shows better prognosis with more endocrine sensitivity and differs from Luminal B tumors, which are more aggressive with less endocrine sensitivity.

### Chromatin Immunoprecipitation

ChIP-on-chip technology could be used to identify the global levels of epigenetically dysregulated genes in tumorigenesis. Thereby, we investigated differentially regulated genes associated with modified H3K9ac and H3K27me3 in breast cancer subtypes. According to their effects on gene regulation, H3K9ac is generally associated with transcriptional activity, while methylation of H3K27me3 is associated with repression^13, 14^. The performance of ChIP experiments was confirmed by using the genes *TSH2B* as a methylated positive control and *C-FOS* as an acetylated positive control. High levels of methylation for *TSH2B* and acetylation for *C-FOS* exhibited the efficiency of immunoprecipitation.

### Epigenetic Dysregulation of Breast Cancer Subtype-Specific Genes

**Figure 1:**
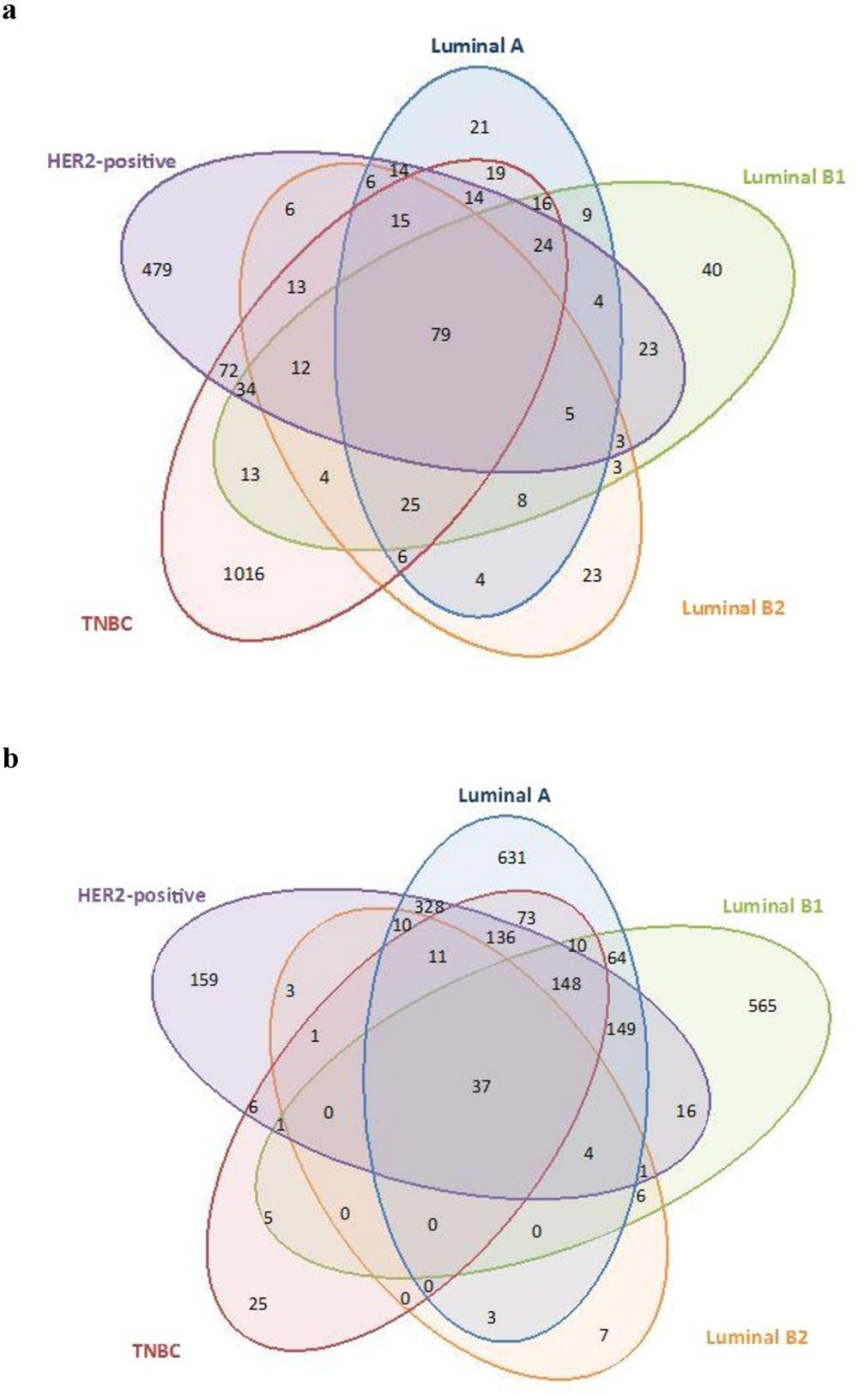
Venn diagrams representing the number of epigenetically modified genes ((**a**) H3K9ac (**b**) H3K27me3) in St Gallen molecular subtypes (luminal A, luminal B1 HER2-negative, luminal B2 HER2-positive, HER2-positive and TNBC) of breast cancer.

**Figure 2:**
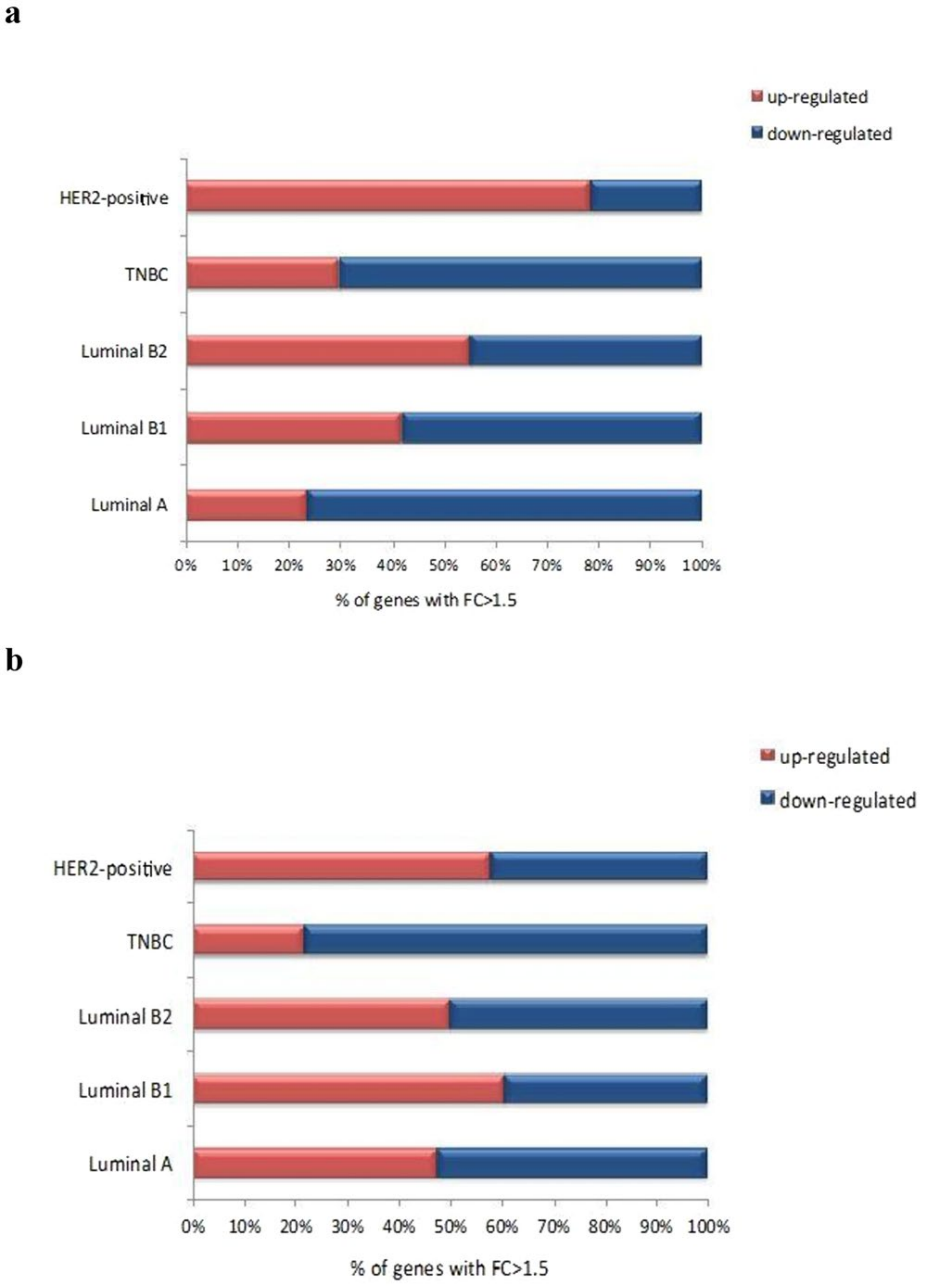
The percentage of epigenetically regulated genes ((**a**) H3K9ac (**b**) H3K27me3) in each breast cancer subtype.

We determined contribution of differentially regulated gene promoters in relation with breast cancer subtypes using Agilent SurePrint G3 Human Promoter microarrays. Venn diagrams were constructed to elucidate the unique and overlapping gene promoters, which were found epigenetically dysregulated between each subtypes (Fig. 1). The aberrant gene regulation associated with H3K9ac modification predominantly was observed in basal-like subtypes; TNBC and HER2-positive tumors. Especially, 1016 modified gene promoters were specific for TNBC population and 479 were unique for HER2-positive tumor samples. H3K9ac epi-mark was down-regulated on gene promoters in HER2-negative tumors (70.1, 76.4 and 58.1% in TNBC, Luminal A and B1 subtype, respectively), beside the percentage of up-regulation found high in HER2-positive tumors (78.5 and 55.2% in HER2-positive, Luminal B2 subtype, respectively, Fig. 2). The level of H3K27me3 was down-regulated on large proportion of promoters in TNBC tumors (78.2%). The enrichment of H3K27me3 epi-mark found higher in tumors with Luminal B1 and HER2-positive subtypes (60.6 and 57.8% in Luminal B1, HER2-positive subtypes, respectively, Fig. 2). According to our results, epigenetic regulations displayed subtype-specific profiles. Notably, the aberrant regulations of genes by H3K9ac epi-mark were common in non-luminal and basal subtype, HER2-positive and TNBC, while modifications in luminal disease, particularly Luminal A and B1, were dysregulated by H3K27me3 epi-mark.

### Breast Cancer Related Network Analysis

**Figure 3:**
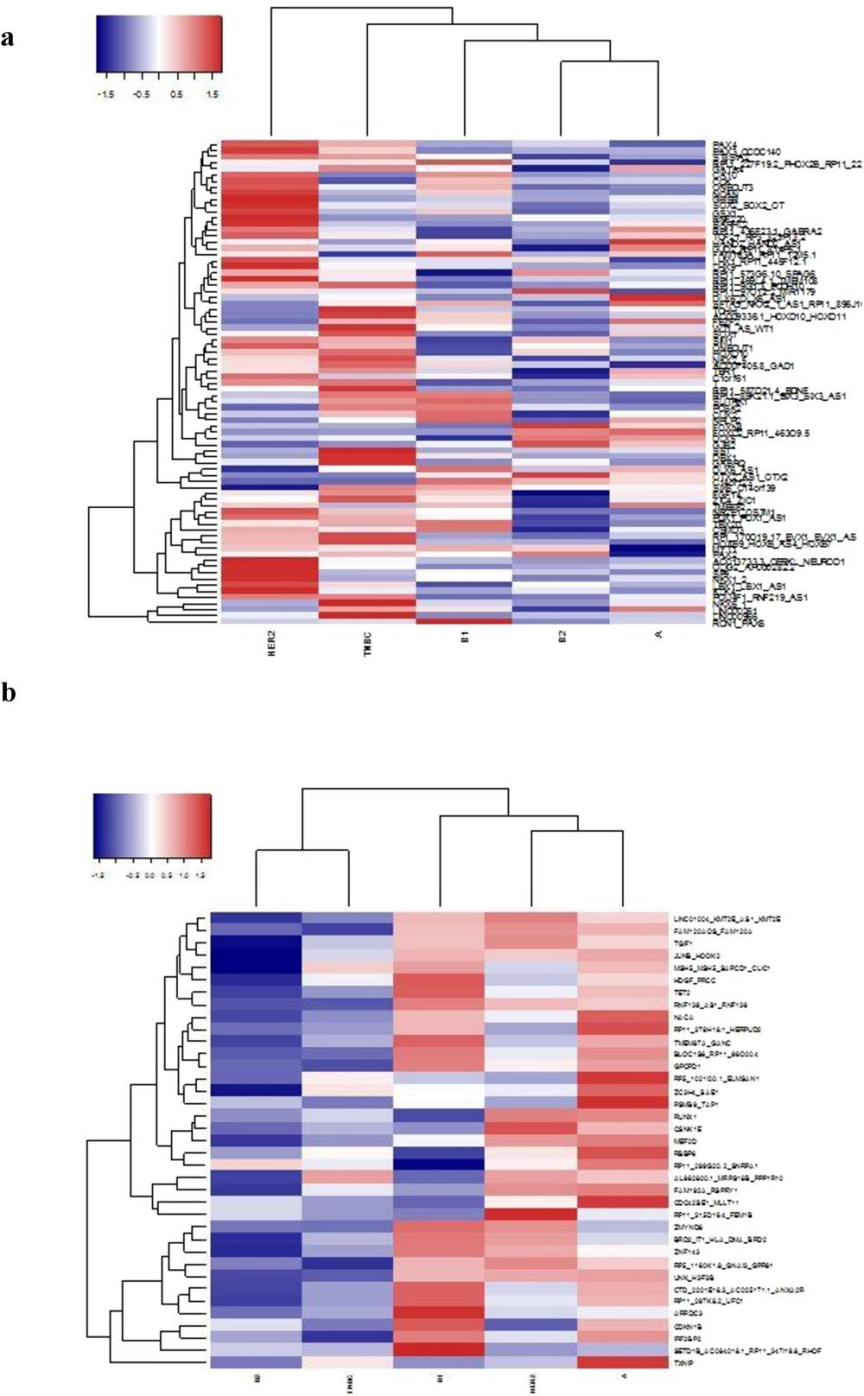
Hierarchical clustering of epigenetically regulated genes in breast cancer tumors. (**a**) H3K9ac-mediated regulation. (**b**) H3K27me3-mediated regulation.

**Figure 4:**
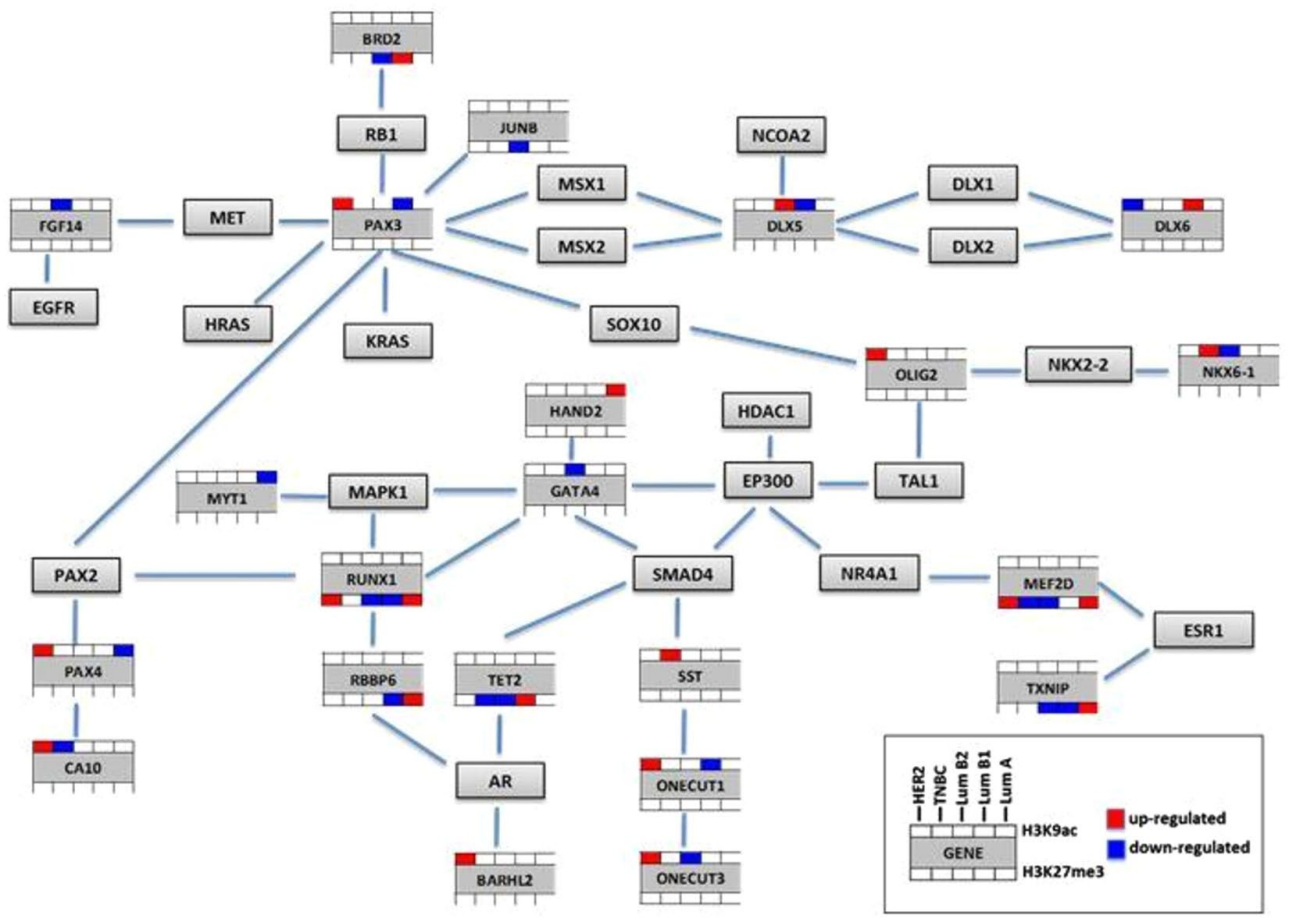
The network structure of differentially regulated genes and nearest-neighbor genes involved in signaling pathways of breast cancer. The network was generated using Cytoscape (version 3.2.0). Epigenetically regulated genes were marked according to their regulation status in different breast cancer subtype.

In this study, an agglomerative method was used: each observation started in its own cluster, and pairs of clusters were merged as one moves up the hierarchy. Two elements were merged in a cluster when their distances were the smallest. The goal of this analysis was to have homogeneous clusters. We identified the prominent dysregulated genes in different breast cancer subtypes using hierarchical clustering data (Fig. 3). The 79 differently regulated genes for H3K9ac epi-mark and 37 genes for H3K27me3 epi-mark were subjected for hierarchical clustering analyses. According to the results of our analyses, the network structure of the genes involved in signaling pathways was generated using Cytoscape program (Fig. 4). The network contained 22 cancer-related target genes; *FGF14*, *PAX3*, *DLX5*, *DLX6*, *MYT1*, *HAND2*, *GATA4*, *OLIG2*, *NKX6-1*, *PAX4*, *CA10*, *BARRHL2*, *SST*, *ONECUT1* and *ONECUT3* differentially regulated by H3K9ac and *RUNX1*, *BRD2*, *JUNB*, *RBBP6*, *TET2*, *MEF2D* and *TXNIP* by H3K27me3. The central genes of the network were *PAX3* (*Paired Box 3*), *DLX5* (*Distal-Less Homeobox 5*), *RUNX1* (*Runt Related Transcription Factor 1*) and *GATA4* (*GATA Binding Protein 4*). *RUNX1* and *PAX3* shown to have oncogene function^15, 16^, while and *GATA4* can be dominantly acting as tumor suppressor genes^17^. *DLX5* contributes to bone development^18^. All those 4 genes were demonstrated to involve in human breast cancer progression.

### Gene Ontology Analysis

We performed Gene Ontology analysis on subtype-specific genes to investigate associated biological process (Table 1). We discovered that H3K9ac modification mainly regulated the biological process of cell proliferation, cell migration, regulation of cell differentiation, negative regulation of programmed cell death and cell-cell signaling. Otherwise, genes regulated by H3K27me3 modification were enriched mostly in cell-cycle regulation, such as negative regulation of cell-cycle, negative regulation of cell-cycle phase transition and cell-cycle arrest. In addition, negative regulation of apoptotic process and negative regulation of apoptotic signaling pathway were remarkable biological process for genes regulated by H3K27me3 epi-mark. The commonly involved biological process revealed that histone 3 modification patterns showed impact on dysregulation of breast cancer-related genes.

**Table 1:** Gene Ontology analysis was performed using DAVID (https://david.ncifcrf.gov/) on H3K9ac-enriched genes in St Gallen molecular subtypes of breast cancer.

GO term name	Luminal A		Luminal B1		Luminal B2		TNBC		HER2-positive	
	up	down	up	down	up	down	up	down	up	down
Regulation of metabolic process	18	44	56	88	49	27	157	498	82	18
Regulation of cell process	22	62	83	121	59	40	217	492	110	9
Cell proliferation	10		24	32	15	10	40	88	28	
Positive regulation of cell proliferation		9	16	17		7			19	
Cell migration	5		18	26	17		28	61	23	
Negative regulation of cell death		10	15	19	8		27	54	19	
Negative regulation of programmed cell death		10	14	17	8		27	52	18	
Regulation of signaling		22	38	38			75	156	40	
Cell-cell signaling	6	17	34	38	13	16	43		32	
Regulation of cell differentiation		16	21	40	20	11	45	79	32	9
Positive regulation of epithelial cell differentiation	2		4		3			6	4	
Hormone-mediated signaling pathway	3			6	4		8			4

The number of differentially modified genes is shown.

## Discussion

In this study, we propounded that distinct breast cancer subtypes show unique epigenetic patterns and epigenetic marks H3K9ac and H3K27me3 figure the post-transcriptional regulation of tumorigenesis processes in breast cancer. We classified 15 breast cancer tumors according to St Gallen Consensus 2013^6^. The epigenetic profiles of H3K9ac and H3K27me3–enriched genes were investigated using ChIP assay coupled with promoter microassay analysis. In particular, the promoter regions of genes modified by H3K9ac epi-mark appeared in HER2-positive and TNBC tumors. The H3K9ac modification induced the down-regulation of the majority of related genes in HER2-amplified tumors. Moreover, H3K9ac-enriched genes were commonly down-regulated in TNBC and Luminal A subtypes. H3K27me3 mark enriched in Luminal A and B1 subtypes and involved in dysregulation of subtype-specific gene expression. The impact of histone modifications on gene regulation patterns has been reported in different breast cancer subtypes^19–21^. However, due to difficulty of working with patient tissue sample, the majority of investigations have been carried out in breast cancer cell lines^21, 22^. Results of these studies asserted that epigenetic modifications are mainly subtype-specific and dysregulation of targeted gene expression profiles result in induction the development of different types of tumor. The high levels of H3K27me3 mark at the promoters of *ER*, *PR* and *ERBB2* genes have been demonstrated in MDA-MB-436 cell lines baring basal-like features^19^. H3K27me3 enrichment was correlated with Luminal A subtype and good prognosis. Likewise, in our study the majority of genes enriched by H3K27me3 epi-mark was included in Luminal A and B1 subtypes. However, Healey *et al*.^23^ reported that global levels of H3K27me3 has been associated with the pathology of Luminal A tumors, no association found with Luminal B subtype, we indicated that H3K27me3 mark was involved in epigenetic regulation of breast tumors with Luminal B1 subtype. Moreover, Holm *et al*.^20^ identified the expression profiles of H3K27me3 in distinct subtypes and the levels of H3K27me3 mark were found diminished in Luminal B, HER2-positive and TNBC tumors, while levels were high in Luminal A tumors. In addition, low abundance of H3K27me3 was propounded as predictor for poor survival. There is limited number of investigations focused on the role of H3K9ac modification on subtype-specific gene expression patterns in breast cancer^22, 24^. Elsheikh *et al*.^24^ have demonstrated the correlation between high levels of H3K9ac mark with better disease-free survival and metastatic-specific survival, and breast cancer- specific survival.

We analyzed differently regulated genes according with histone modification patterns and 79 genes became prominent for H3K9ac and 37 genes for H3K27me3 mark. Afterwards, targeted genes for breast cancer development were distinguished and network analysis was constructed. We observed a strong H3K9ac signal in promoters of *FGF14*, *PAX3*, *DLX5*, *DLX6*, *MYT1*, *HAND2*, *GATA4*, *OLIG2*, *NKX6-1*, *PAX4*, *CA10*, *BARRHL2*, *SST*, *ONECUT1* and *ONECUT3*; *RUNX1*, *BRD2*, *JUNB*, *RBBP6*, *TET2*, *MEF2D* and *TXNIP* for H3K27me3. The central genes of the network were identified and *RUNX1*, *PAX3*, *GATA4* and *DLX5* genes were subjected for epigenetically dysregulation in association with diversity of breast cancer subtypes.

The *RUNX* family genes are transcription factors and take part in hematopoiesis, osteogenesis and neurogenesis^25^. *RUNX1* involves in tumorigenesis as a key regulatory factor, particularly in various epithelial cancers^26, 27^. According to our results, H3K27me3 enrichment was up-regulated on *RUNX1* gene in HER2-positive and Luminal A subtype, however down-regulation was determined in tumors with Luminal B1 and B2 subtype. A previous study indicated that *RUNX1* conducted as oncogene in TNBC and the expression levels of *RUNX1* was correlated with the poorest prognosis^28^. It is propounded that the effect of *RUNX1* on tumor progression was conducted with its ability to diminish *ER* signaling. We did not found any correlation between *RUNX1* regulation by H3K27me3 mark and TNBC subtype.

*PAX3* gene is acting as an oncogene in breast tumorigenesis and the expression profile of *PAX3* gene is regulated by epigenetic mechanisms. Recently, Zhao *et al*.^16^ asserted that reduced level of H3K9me2 epi-mark in primary breast epithelial cells was involved in regulation of *PAX3* expression. In our study, we demonstrated that H3K9ac enrichment increased on *PAX3* oncogene in HER2-positive tumors. Hence, non-luminal HER2-positive tumors had relatively low five-year related survival and poorer prognoses than Luminal A, the epigenetic dysregulation of *PAX3* by H3K9ac come forward as a pathological prognostic factor for HER2-positive tumors.

The differently expression of transcription factor *GATA4* has been reported in ERBB2-amplified breast tumors^29^. The role of *GATA4* in epigenetic mechanisms is not clarified, however it has been shown that *GATA4* possessed *ERBB2* gene expression through transcriptional repression. The epigenetic regulation of *GATA4* gene by H3K9ac modification has been shown in Luminal B and HER2-amplified tumors. On the other hand, we could not contribute to the H3K9ac levels with non-luminal HER2-positive tumors.

*DLX5* gene is mainly expressed in developing bones and controls osteoblastogenesis^30^. In addition, the overexpression has been observed in more aggressive tumors and increased risk of metastases in bone or lung^18^. The H3K9ac-enrichment on *DLX5* gene was identified in Luminal B2 subtype in this study. The epigenetic dysregulation of *DLX5* gene could be suggested as an indicator for aggressiveness of breast tumors.

According to GO term analysis on subtype-specific genes, H3K9ac modification was commonly involved in biological process of cell proliferation, cell migration, regulation of cell differentiation, negative regulation of programmed cell death and cell-cell signaling. On the other hand, the genes differently regulated by H3K27me3 mark were associated with biological process of negative regulation of cell-cycle, negative regulation of cell-cycle phase transition and cell-cycle arrest. Moreover, negative regulation of apoptotic process and negative regulation of apoptotic signaling pathway were remarkable biological processes for genes regulated by H3K27me3 epi-mark. It is not surprising that all these biological processes involve in the hallmark of cancer. However, shared biological processes vary across histone marks.

In this study, epigenetic modifications H3K9ac and H3K27me3 were analyzed to identify the role of epigenetic mechanisms on diversity of subtype-specific gene regulation in breast cancer. Tumor size, histological subtype and grade, lymph node status and expression of ER, PGR and HER2 are routinely used for classification of breast cancer tumor. However, these parameters are restrictive to predict individual survival and response to therapy. Our results may provide knowledge about subtype-specific epigenetic regulations in breast cancer. Mainly, expression profiles demonstrate diversity in HER2 positive and basal-like breast tumors. Working with tumor samples restricted our study because of difficulty of sample enrichment, cell heterogeneity and individual variations. New methodological improvements in epigenetic researches such as ChIP-on-chip methods would lead to a better understanding of underlying mechanisms of breast tumorigenesis and provide new approaches for prevention and treatment with clarifying the role of additional mechanisms and complex epigenetic regulations.

## Methods

### Patients

The study was carried out with 15 human breast cancer tumors and matched normal tissue samples. The samples were obtained from a Biological Resources Center (BB‐0033‐00075) and a prior signed informed consent was obtained from each patient. It was done in accordance with the Council of Europe’s Recommendation on Research on Biological Materials of Human Origin [Rec(2006)4] from 2006^31^. Patients who had undergone neoadjuvant chemotherapy, hormone therapy, radiotherapy and family history of breast cancer were excluded. Breast cancer subtypes were classified according to St Gallen International Expert Consensus 2013, related with their clinical profiles and hormone receptor status^6^. All experiments and methods were performed in accordance with relevant guidelines and regulations.

### Chromatin Immunoprecipitation (ChIP)

DNA extraction and shearing procedures were performed as indicated previously^32, 33^. ChIP was carried out with SX-8G IP-Star® Compact Automated System (Diagenode). Anti-H3K27me3 (pAb-069-050), anti-H3K9ac (pAb-103-050) and non-immune rabbit IgG (Kch-504-250) were obtained from Diagenode. The performance of ChIP experiments was confirmed by Q-PCR (ABI PRISM 7900HT, Applied Biosystems). Primer sequences of human gene *TSH2B* (Diagenode) were used as positive control for methylation and *C-FOS* (Diagenode) for acetylation. The experimental protocol was conducted according to^32, 33^.

### Microarray Hybridization and ChIP-on-chip

Human Promoter Microarrays were provided by Agilent Technologies to determine the histone methylation or acetylation of ChIP-enriched samples (SurePrint G3 400k Human Promoter microarrays). The array contains ~21,000 of the best-defined human gene regions. Hybridization and microarray procedures were performed in accordance with the manufacturer’s instructions. Each microarray analysis was duplicated for one tumor and matched normal tissue samples. A High Resolution Microarray Scanner (Agilent Technologies) was utilized for scanning fluorescent intensities.

### Microarray Data Analysis

ChIP-on-chip data analyses were carried out using the package R/Bioconductor Ringo software 1.26.1. The selection criteria for the enriched regions or genes were: the region require a certain 250 bp probe spacing; contain at least 3 probes/region and smoothed intensities of probes mapped to this region need to exceed a threshold defined at 1.5.

### Network Analysis

The network associated with genes regulated by epigenetic modifications among different subtypes was constructed using Cytoscape^14^ (version 3.2.0). Particularly, cancer-related target genes and nearest-neighbor genes were considered to build the network structure.

### Functional Annotation

Gene Ontology biological process terms analysis in breast cancer subtype-specific histone modification associated genes were determined using DAVID Functional Annotation Tool^34^.

## References

[1] Prat A, Perou CM (2011). Deconstructing the molecular portraits of breast cancer. Mol Oncol.

[2] Bauer K, Parise C, Caggiano V (2010). Use of ER/PR/HER2 subtypes in conjunction with the 2007 St Gallen Consensus Statement for early breast cancer. BMC Cancer.

[3] Slamon DJ (1987). Human breast cancer: correlation of relapse and survival with amplification of the HER-2/neu oncogene. Science.

[4] Chin K (2006). Genomic and transcriptional aberrations linked to breast cancer pathophysiologies. Cancer Cell.

[5] Goldhirsch A (2011). Strategies for subtypes–dealing with the diversity of breast cancer: highlights of the St. Gallen International Expert Consensus on the Primary Therapy of Early Breast Cancer 2011. Ann Oncol.

[6] Goldhirsch A (2013). Personalizing the treatment of women with early breast cancer: highlights of the St Gallen International Expert Consensus on the Primary Therapy of Early Breast Cancer 2013. Ann Oncol.

[7] de Azambuja E (2007). Ki-67 as prognostic marker in early breast cancer: a meta-analysis of published studies involving 12,155 patients. Br J Cancer.

[8] Inic Z (2014). Difference between Luminal A and Luminal B Subtypes According to Ki-67, Tumor Size, and Progesterone Receptor Negativity Providing Prognostic Information. Clin Med Insights Oncol.

[9] Trihia H (2003). Ki-67 expression in breast carcinoma: its association with grading systems, clinical parameters, and other prognostic factors–a surrogate marker?. Cancer.

[10] Cheang MC (2009). Ki67 index, HER2 status, and prognosis of patients with luminal B breast cancer. J Natl Cancer Inst.

[11] Dworkin AM, Huang TH, Toland AE (2009). Epigenetic alterations in the breast: Implications for breast cancer detection, prognosis and treatment. Semin Cancer Biol.

[12] Judes G (2016). H3K4 acetylation, H3K9 acetylation and H3K27 methylation in breast tumor molecular subtypes. Epigenomics.

[13] Martin C, Zhang Y (2005). The diverse functions of histone lysine methylation. Nat Rev Mol Cell Biol.

[14] Shannon P (2003). Cytoscape: a software environment for integrated models of biomolecular interaction networks. Genome Res.

[15] Barutcu AR (2016). RUNX1 contributes to higher-order chromatin organization and gene regulation in breast cancer cells. Biochim Biophys Acta.

[16] Zhao QY (2016). Global histone modification profiling reveals the epigenomic dynamics during malignant transformation in a four-stage breast cancer model. Clin Epigenetics.

[17] Natrajan R (2012). A whole-genome massively parallel sequencing analysis of BRCA1 mutant oestrogen receptor-negative and -positive breast cancers. J Pathol.

[18] Morini M (2010). Mutually exclusive expression of DLX2 and DLX5/6 is associated with the metastatic potential of the human breast cancer cell line MDA-MB-231. BMC Cancer.

[19] Chen X (2016). A novel subtype classification and risk of breast cancer by histone modification profiling. Breast Cancer Res Treat.

[20] Holm K (2012). Global H3K27 trimethylation and EZH2 abundance in breast tumor subtypes. Mol Oncol.

[21] Li Y (2014). Comparative epigenetic analyses reveal distinct patterns of oncogenic pathways activation in breast cancer subtypes. Hum Mol Genet.

[22] Hong CP, Choe MK, Roh TY (2012). Characterization of Chromatin Structure-associated Histone Modifications in Breast Cancer Cells. Genomics Inform.

[23] Healey MA (2014). Association of H3K9me3 and H3K27me3 repressive histone marks with breast cancer subtypes in the Nurses’ Health Study. Breast Cancer Res Treat.

[24] Elsheikh SE (2009). Global histone modifications in breast cancer correlate with tumor phenotypes, prognostic factors, and patient outcome. Cancer Res.

[25] Speck NA, Terryl S (1995). A new transcription factor family associated with human leukemias. Crit Rev Eukaryot Gene Expr.

[26] Hoi CS (2010). Runx1 directly promotes proliferation of hair follicle stem cells and epithelial tumor formation in mouse skin. Mol Cell Biol.

[27] Yeh HY (2009). Identifying significant genetic regulatory networks in the prostate cancer from microarray data based on transcription factor analysis and conditional independency. BMC Med Genomics.

[28] Ferrari N (2014). Expression of RUNX1 correlates with poor patient prognosis in triple negative breast cancer. PLoS One.

[29] Hua G (2009). A negative feedback regulatory loop associates the tyrosine kinase receptor ERBB2 and the transcription factor GATA4 in breast cancer cells. Mol Cancer Res.

[30] Merlo GR (2000). Multiple functions of Dlx genes. Int J Dev Biol.

[31] Lwoff L (2008). Ethics of research on human biological materials. Nature biotechnology.

[32] Dagdemir A, Durif J, Ngollo M, Bignon YJ, Bernard-Gallon D (2013). Histone lysine trimethylation or acetylation can be modulated by phytoestrogen, estrogen or anti-HDAC in breast cancer cell lines. Epigenomics.

[33] Dagdemir A (2016). Epigenetic Modifications with DZNep, NaBu and SAHA in Luminal and Mesenchymal-like Breast Cancer Subtype Cells. Cancer Genomics Proteomics.

[34] Huang da W, Sherman BT, Lempicki RA (2009). Systematic and integrative analysis of large gene lists using DAVID bioinformatics resources. Nat Protoc.

